# Docetaxel-Induced Systemic Sclerosis with Internal Organ Involvement Masquerading as Congestive Heart Failure

**DOI:** 10.1155/2017/4249157

**Published:** 2017-02-06

**Authors:** Bumsoo Park, Raghavendra C. Vemulapalli, Amit Gupta, Maria E. Shreve, Della A. Rees

**Affiliations:** Department of Family Medicine, Henry Ford Hospital, Wayne State University School of Medicine, Detroit, MI 48202, USA

## Abstract

Systemic sclerosis, or scleroderma, is a complex medical disorder characterized by limited or diffuse skin thickening with frequent involvement of internal organs such as lungs, gastrointestinal tract, or kidneys. Docetaxel is a chemotherapeutic agent which has been associated with cutaneous side effects. An uncommon cutaneous side effect of docetaxel is scleroderma-like skin changes that extend from limited to diffuse cutaneous systemic sclerosis. Several case reports have been published regarding the association of docetaxel and systemic sclerosis. However, those reports demonstrated the association between docetaxel and scleroderma-like skin changes without internal organ involvement. Here, we report a case of systemic sclerosis with pulmonary arterial hypertension and a microangiopathic kidney involvement induced by docetaxel chemotherapy. After an exhaustive literature review, this could be the first case of docetaxel-induced systemic sclerosis involving internal organs.

## 1. Introduction

Systemic sclerosis, or scleroderma, is a heterogeneous disorder characterized by skin sclerosis as the baseline pathology. Systemic sclerosis is classified into three subsets: limited cutaneous systemic sclerosis, diffuse cutaneous systemic sclerosis, and systemic sclerosis without skin involvement [1]. The limited subset typically involves skin sclerosis that is restricted to hands or, to a lesser extent, to the face and neck, whereas the diffuse subset involves skin thickening extending to either proximal extremities or trunk and has demonstrated a higher frequency of internal organ involvement (lungs, gastrointestinal tract, or kidneys).

Docetaxel is a chemotherapeutic agent as a member of taxane drug class. Well-known side effects of docetaxel include peripheral neuropathy, myelosuppression, arthralgia, myalgia, fluid retention, and cutaneous reactions. One uncommon cutaneous side effect of docetaxel is a scleroderma-like skin change for which there have been more than 10 case reports in the literature. However, the side effects that all of the case reports have shown were scleroderma-like skin change, or limited/diffuse cutaneous systemic sclerosis, without providing evidence of internal organ involvement.

Herein we report a case with docetaxel-induced systemic sclerosis with internal organ involvement masquerading as a congestive heart failure (CHF). After an exhaustive literature review, this could be the first case of docetaxel-induced systemic sclerosis involving systemic organs.

## 2. Case Report

A 49-year-old African American woman presented with shortness of breath, who was discharged from the other hospital one week earlier with the diagnosis of acute exacerbation of CHF. Patient was given oral furosemide and carvedilol at discharge, however, she admitted to losing her prescription for carvedilol resulting in her not taking it since discharge. She exhibited bilateral leg edema on examination without crackles or jugular venous distention. Brain natriuretic peptide was elevated to 1330 pg/mL. Chest radiograph demonstrated cardiomegaly with minimal interstitial edema. Echocardiography from the other hospital showed an ejection fraction (EF) of 50%, pulmonary arterial pressure (PAP) of 56 mmHg, and normal left ventricular diastolic filling. Recurrent exacerbation of CHF secondary to idiopathic pulmonary arterial hypertension (PAH) triggered by medication noncompliance was initially diagnosed, and the patient was started on intravenous furosemide. However, no clinical improvement was noted with no reasonable urine output. The medical team doubted the diagnosis of CHF exacerbation and started to do more thorough history taking and a comprehensive physical examination.

**Figure 1:**
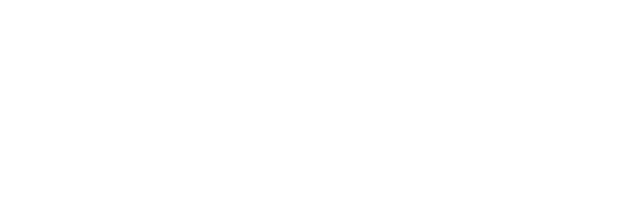
Diffuse skin thickening and sclerosis of bilateral hands that cross the metacarpophalangeal joint.

The patient had a history of breast cancer and underwent left lumpectomy followed by 4 cycles of chemotherapy using docetaxel/cyclophosphamide and radiation therapy about a year ago. She was given 134 mg of docetaxel and 1,074 mg of cyclophosphamide on a monthly basis. On repeat examination, she demonstrated diffuse thickening/sclerosis of bilateral hands that crossed metacarpophalangeal joints extending up to bilateral proximal upper extremities (Figure 1). Extensive sclerosis of skin and subcutaneous tissue of anterior chest wall and the left breast were also noted. Patient endorsed that she started to have pain and stiffness of both hands right after she finished the last cycle of chemotherapy. Echocardiography before the chemotherapy showed EF of 55%, PAP of 26 mmHg, and normal left ventricular (LV) diastolic filling. LV size has been normal without change before and after the chemotherapy. Therefore, her estimated systolic PAP has increased by more than 2-fold after the chemotherapy with newly developing skin sclerosis. Docetaxel-related systemic sclerosis was suspected. Serologic test showed positive antinuclear antibody, but negative anticentromere and anti-Scl-70 antibodies. Pulmonary function test was normal with slightly decreased diffusing capacity. High-resolution computed tomography of the chest did not show interstitial lung disease. Finally, it was noted that she met the 2013 American College of Rheumatology Classification Criteria of Systemic Sclerosis as she demonstrated skin thickening of the fingers of both hands extending proximal to the metacarpophalangeal joints (score 9) and PAH (score 2). Per the criteria, patients with a total score of ≥9 are classified as having definite systemic sclerosis [2].

We discontinued diuretic treatment and continued on beta-blocker with calcium-channel blocker. Patient's clinical condition improved and was discharged with follow-up appointment with Rheumatology and Pulmonary Hypertension (PH) clinic.

Upon follow-up chart review, the patient is currently following Rheumatology, Pulmonology, and Dermatology but is also on hemodialysis given worsening renal function with following Nephrology. Renal biopsy was performed and it showed thrombotic microangiopathy pattern (classic “onion-peeling” appearance) which is suggestive of scleroderma per pathologic documentation. She was diagnosed with scleroderma renal crisis by Nephrology and was started on captopril. Patient is currently stable with normal blood pressure and stable serum creatinine levels.

## 3. Discussion

Our case showed newly developed PAH, thrombotic microangiopathy of the kidney with scleroderma renal crisis, and skin sclerosis of bilateral hands definitive of systemic sclerosis, status post docetaxel treatment.

Among side effects of docetaxel, it has been reported that docetaxel can induce a variety of skin toxicities [3]. One of the rare cutaneous side effects of docetaxel is a scleroderma-like change, and there have been more than 10 case reports. Since three cases of limited systemic sclerosis induced by docetaxel were reported at first [4], there have been reports regarding both limited cutaneous systemic sclerosis [5, 6] and diffuse cutaneous systemic sclerosis [7, 8] induced by docetaxel. The exact mechanism by which docetaxel induces skin sclerosis is unclear. Versican is a large chondroitin sulfate proteoglycan expressed by human fibroblasts. It interacts with cells and enables them to proliferate, migrate, adhere, and assemble an extracellular matrix [9]. Hesselstrand et al. suggested that versican may contribute to skin thickening with excess collagen deposits in systemic sclerosis [10]. Okada et al. reported that intense versican deposits were found in the skin after docetaxel administration [11]. Therefore, versican may play a role in pathogenesis of docetaxel-induced systemic sclerosis.

One of the interesting findings in our report is that our patient developed PAH after docetaxel treatment. So far, no cases have been reported associating docetaxel with PAH. It has been hypothesized that PAH in systemic sclerosis occurs as a consequence of progressive remodeling of pulmonary vasculature with inflammation and endothelial injury as common precursors. A recent study revealed that docetaxel facilitates endothelial injury through oxidative stress mechanism [12]. Thus, docetaxel may induce PAH either by oxidative injury to the endothelial cells or by stimulating fibroblast cells as mentioned earlier.

PH can also be developed by LV dysfunction (Group 2 PH), and it is possible that the patient might have had Group 2 PH given decreased EF from 60% to 50%. However, based on the fact that estimated systolic PAP increased by more than 2-fold compared to decrease in EF by 17% and the LV diastolic function remained normal before and after chemotherapy, it is more likely that the patient might have developed primary PAH (Group 1 PH). Another possible consideration is that cyclophosphamide which the patient was given can cause dilated cardiomyopathy, and this condition might have caused Group 2 PH. However, given the fact that LV size remained constantly normal before and after chemotherapy, it is less likely that the patient developed dilated cardiomyopathy after cyclophosphamide treatment.

It should also be considered that systemic sclerosis might have developed as a manifestation of breast cancer. Joseph et al. have suggested that a tumor antigen can trigger autoimmune response which can develop systemic sclerosis after they found that a certain genetic mutation triggered cellular immunity and cross-reactive humoral immune responses [13]. However, based on the fact that the patient started to have skin thickening after she finished the chemotherapy and she was diagnosed with breast cancer 2 years ago, it is more likely that the patient developed systemic sclerosis secondary to chemotherapy.

Our study has a limitation that the PAH was diagnosed only based on echocardiography, but not confirmed by right heart catheterization (RHC). RHC should be performed to diagnose PAH in systemic sclerosis per 2013 American College of Rheumatology Classification Criteria of Systemic Sclerosis. We deferred RHC while the patient was hospitalized since the patient was too weak and fatigued to tolerate invasive procedures. However, as the patient also demonstrated biopsy-proven microangiopathic kidney involvement which was pertinent to systemic sclerosis, we still believe that the patient developed an internal organ involvement of systemic sclerosis with not only having skin involvement.

We also admit that we initially missed characteristic findings of bilateral hand sclerosis on examination because we focused only on assessing cardiopulmonary status. An observational study showed that 26% of the inpatient sample had diagnosis and treatment changed substantially as a result of the attending physician's physical examination [14]. Therefore, it cannot be overemphasized that a thorough history taking and comprehensive physical examination are essential for medical practice.

## 4. Conclusion

The association between docetaxel and scleroderma-like skin changes has been well established in the literature. However, we firstly report a case that docetaxel can also cause systemic sclerosis with internal organ involvement that manifested as PAH and microangiopathic kidney disease. Therefore, we may need to consider the possibility of systemic sclerosis and try to look for any skin thickening if a patient develops unexplained PAH or worsening renal function with docetaxel treatment.
