# Corrigendum to “Docetaxel-Induced Systemic Sclerosis with Internal Organ Involvement Masquerading as Congestive Heart Failure”

**DOI:** 10.1155/2017/4809124

**Published:** 2017-06-29

**Authors:** Bumsoo Park, Raghavendra C. Vemulapalli, Amit Gupta, Maria E. Shreve, Della A. Rees

**Affiliations:** Department of Family Medicine, Henry Ford Hospital, Wayne State University School of Medicine, Detroit, MI 48202, USA

In the article titled “Docetaxel-Induced Systemic Sclerosis with Internal Organ Involvement Masquerading as Congestive Heart Failure” [1], Figure 1 is blanked due to the lack of written consent from the patient.
